# ‘Standing on the Shoulders of the Giants’: Dr Rainer Storb

**DOI:** 10.1007/s44228-022-00018-2

**Published:** 2022-08-29

**Authors:** Mohamad Mohty, Rainer Storb

**Affiliations:** 1 Department of Clinical Hematology and Cellular Therapy, Saint-Antoine Hospital, AP-HP, Sorbonne University, Paris, France; 2 Sorbonne University, INSERM, Saint-Antoine Research Centre, Paris, France; 3 Division of Clinical Research, Fred Hutchinson Cancer Research Center, University of Washington, Seattle, WA USA; 4 Department of Medicine, Fred Hutchinson Cancer Research Center, University of Washington, Seattle, WA USA

**Keywords:** Stem cell transplantation, Graft versus host disease, Conditioning

In a letter to Robert Hooke in 1675, Sir Isaac Newton made his most famous statement: *“if I have seen further, it is by standing on the shoulders of giants”.* This initiative of the International Academy for Clinical Hematology (IACH) aims to celebrate the achievements of leading experts and investigators whose work and research have helped to significantly advance the field of clinical hematology and establish the milestones and foundations of modern clinical hematology. The IACH Scientific Steering Committee will choose the honorees from different fields of clinical hematology. The choice is based on the nominee’s body of work including strength of research, clinical impact, significant educational contributions, and overall accomplishments. Our first honoree is Dr. Rainer Storb. This report represents a transcript of the interview given by Dr. Rainer Storb (RS) on the 17th of February 2022 who responded to a series of questions asked by Dr. Mohamad Mohty (MM).

MM: Good morning, Dr. Storb. Everything has been said about your great achievements and we owe you a lot, but my first question is, I wonder if you can tell us about your early childhood as everyone has spoken about transplantation and total body irradiation etc. Your early educational experience and how that may have played a role in your selection of scientific and medical fields?

RS: Well, actually I’m not entirely sure whether my early childhood contributed. I grew up in fairly turbulent times, WW II, the post-war period. During the war I grew up in a city that was heavily bombed, and schools were all destroyed in the process and so I was put in a small boarding school in the Bavarian Alps, separated from my parents for quite a while. When I came back, cities were destroyed, we didn’t have school for the first year after the war because there were no buildings, so I’m not sure that that actually contributed to the choice. I was just innately very curious about things and maybe that was the motivating factor. When I was 19 years old and finishing Gymnasium (sort-of the equivalent of high school), my choices were engineering which fascinate me, or medicine which was a family tradition. My grandfather was a surgeon, and my father was a general practitioner who had served as a medic during WW I in the Russian front and then later established his practice. So, it may have been the family tradition that motivated me to go into medicine but then, when I was in medicine, I recognized that I wasn’t really cut out for the daily routine of seeing patients. I was becoming really curious about what was behind a disease or disease phases and what was behind therapy, so I think that was what drove me into the medical sciences.

MM: Was there any particular teacher or faculty in Germany or elsewhere that you thought at the time to be exemplary of the academic tradition and these guys inspired you to push you further?

RS: Well, I did my last phases of my medical education at the University of Freiburg, in southwest Germany and there, hematology was very much in the foreground. There was a very charismatic teacher, Ludwig Heilmeyer, who stimulated my interest in hematology especially the bone marrow and that’s what motivated me to choose an internship and residency in Munich with another hematologist named Herbert Begemann. Neither one was a laboratory-based physician, but they definitely had a great interest in hematologic diseases. When I looked at postdoctoral fellowships, I looked at Paris and I applied for a NATO Science Fellowship which I obtained to work first at the Hôpital Saint-Louis under Jean-Bernard on the more clinically oriented side and then I went to work with Marcel Bessis at the National Transfusion Center which was laboratory-based research and that’s where I became really interested in basic laboratory-based research. My focus was always on laboratory-based research which could be translated into helping patients. So, it was actually the stay in Paris which shaped my future outlook.

MM: Well, I’m very pleased to hear this and it is really very exciting but what was it that was happening in Seattle actually, that attracted you to move there from Paris?

RS: Well, while I was in Paris, I realized that I didn’t want to be devoting my life entirely to laboratory-based research and while I was working with Marcel Bessis, we had a lot of visitors to the lab from all over the world and I used the opportunity to speak to them. It was very obvious that the only country at that time where lab work could be connected to research was the USA. A German investigator named Theodor (Ted) Fliedner had worked for nine years at the Brookhaven National Laboratories on Long Island with Eugene Cronkite. When he visited the Bessis lab he was on his way back to Germany (where he established a research facility at the University of Ulm), he recommended that I contact three people for a postdoctoral stay: one was Fred Stohlman in Boston, one was George Brecher at the National Institutes of Health (NIH), Bethesda, and then he said the one that was really interesting was E. Donall (Don) Thomas who had just moved to Seattle. I looked at their publications in the library and I thought that Don was the most interesting guy. I wrote to all of them, and they all offered space in their lab. I applied for a Fulbright scholarship and decided in the end to work with Don in Seattle. I had no idea where Seattle was, to be frank, I thought it was the East Coast of the USA. My choice to go to Seattle was strengthened by an experience I had in 1964 with George Mathé, whom I knew. He had set up a research conference on bone marrow transplantation where I met people like Dirk van Bekkum from Holland and, of course George Mathé. I met Ernie McCulloch from Toronto, Emil Freireich from the NIH, Jacques Miller from Melbourne, Robert Good from Minneapolis, and D. W. H. Barnes, C. E. Ford, and J. F. Loutit from the Harwell laboratories in the UK and I was fascinated by the discussions these people were having, heated discussions and disagreements on how the field would evolve and this made me go to Don Thomas in Seattle, even though at the time, human bone marrow transplantation seemed to have failed. In fact, most people who were working in the field were dismayed, thinking this would never really become a medically useful procedure. This was what fascinated me and so with the help of the Fulbright scholarship, in September 1965, I finally made my way to the west coast of the USA.

MM: Today, when we speak about bone marrow transplantation, it is part of hematology. So, what was the structure of the hematology field back then in the 60 s when you arrived in Seattle? Was there an actual department of hematology at that time; did anyone call themselves a hematologist?

RS: Yes, in Seattle there was the division of hematology, led by the then giant of hematology, the “iron man” Clem Finch who worked in iron metabolism and he helped setting up a small sub-division of hematology for Don Thomas. Clem was the one who recruited Don Thomas to Seattle from Cooperstown, New York, to head this sub-division and the letterhead was supposed to say, *“Division of Hematology and Transplantation”* but the University of Washington printing office made a mistake, calling it *“Division of Hematology and Transportation”,* so unknown was this field at that time. Don’s division comprised Don himself, a full professor, there was an instructor in medicine named Robert Epstein, who became a really good friend and colleague. I was the senior research fellow and we had one secretary, Louise, and a laboratory technician, Kathy, and two animal technicians, one of whom was Ted Graham, an ex-marine sergeant who had travelled with the Thomas family from Cooperstown, New York to Seattle, and that was the Division of Hematology and Transportation! We weren’t even located at the University of Washington campus but in a teaching hospital called the United States Public Health Service Hospital, which was 6.8 miles south of the medical school and was an old hospital from the 1930s. We had a small laboratory up on the 10th floor consisting of two lab benches, a small office, and an animal laboratory. There was only one other research unit in the building, that of Seymour Klebanoff, who became well known for his work on how granulocytes kill bacteria. We were very isolated and even a trip to the library was 6.8 miles away in the medical school. So that’s how we started, very primitively.

For most of our animals we were given space in an abandoned WW II bunker which was another 5 miles away in West Seattle, up on a hillside above the port. We had to go down five flights of stairs underground to enter the bunker where we had this small animal breeding facility and where we had the total body irradiation sources. We were actually the division of transportation because we had to drive all the time to get to our laboratory spaces or the library and if you wanted to attend seminars at the medical school.

MM: I heard clearly that the division of hematology was focused on iron metabolism.

RS: Correct.

MM: Then you had this sub-division of transportation where you were trying to find your way and maybe this can explain your keenness for physical activity and sport. Can you talk a little on the sort of research and clinical problems that people were debating in the field of blood and bone marrow disease at this time?

RS: As I mentioned, the human transplantation efforts had been largely abandoned when I came to Seattle, no one was doing human transplants. There was our small group, and another equally small group under George Santos, working with rats at the Johns Hopkins University in Baltimore. There was George Mathé in Paris who was highly innovative, but I think, not all that well organized and Dirk van Bekkum at the Rijswijk Radiation Biology Institute working with monkeys. There were some individuals in the UK at the Harwell Laboratories. Barnes, Ford, and Loutit worked with mice, and John Trenton in Houston, and there were several people at the Oak Ridge National Laboratory, Charlie Congdon, Egon Lorenz, Nazareth Gengozian, T. Makinodan, and Delta Uphoff. These were the people working in the field, except for George Santos and us, nobody really took an organized approach working in the lab and hoping to apply the lab work to human patients. Our interests were patients with leukemia, aplastic anemia, and hemoglobinopathies. So, when I first arrived, Bob Epstein and I made a list of things that had gone wrong in the preceding attempts at human transplantation, starting with conditioning regimens and tissue typing which was back then, a big black box. The Europeans played a big role in unravelling it eventually, like Jean Dausset in Paris and Jon van Rood in Leiden. We then looked at GvHD prevention, we were concerned about the aplastic anemia patients becoming sensitized to their donors via transfusions, so we then began animal experimentation systematically to work out these problems. It took 5 years of intensive animal work before anything got into the clinic. We wrote to veterinarians in the western USA to send us dogs with spontaneous lymphoma (with consent from their owners) and said we would treat them with either autologous or allogeneic transplants and send them back to their owners. This was a very helpful undertaking because we learned about autologous transplantation and graft-*versus*-tumour (GvT) effects which up to that time had only been described once in mice in a 1956 publication by Barnes and Loutit. These two investigators didn’t really know whether it was a GvT effect or whether the GvHD seen in these mice provided an infertile ground for leukemia development. This was one of the questions that we tried to answer more definitively.

We worked alone more or less with not much support from colleagues in Seattle who thought we were idiots pursuing a line of research that was doomed to fail!

I remember very distinctly in 1966, I happened to be sitting next to the Department Chair of Immunology in Seattle, Russ Weiser, on a flight to the transplantation meetings in New York, and for the whole trip he kept telling me that transplantation would never come to pass, and we would never be able to cross the barrier from one individual to another, so our future seemed dim. Sir Peter Medawar, a Nobel Laureate, told me at the meeting that the barrier from one individual; to another could never be crossed.

MM: But you made it! Which fields outside of hematology would have most influenced your thinking within your field back then? For instance, were there more fashionable discussions on other breakthroughs or developments through seminars/conferences?

RS: Well, you know, we only had funds to attend one research meeting per year so we either chose to attend the transplantation meeting, the American Hematology Society meeting or the Experimental Hematology Society meeting. What really influenced us were developments in tissue typing so we had contact very early on, especially with Jan van Rood in Leiden, and Bob Epstein spent a few months there. Then we focused on dogs, developing DLA (dog leukocyte antigen) typing technology which was very primitive at the time. We spent an inordinate amount of time on creating antisera by cross-immunizing littermate dogs and then taking these antisera and the trypan blue exclusion assay developed by Paul Terasaki in Los Angeles, and doing systematic studies of pedigrees of animals which turned out to be a disaster because of the fickleness and inaccuracy of the testing system.

Eventually in 1967, before tossing the frustrating antibodies into the sink, we decided to do one last experiment. It took us two hours to persuade Don Thomas to buy us the animals, 6 pairs of littermates, from which we picked a donor from each pair, then one recipient that was unequally matched and one that was unequivocally mismatched by the serological reactions. We coded the numbers of these animals, the secretary kept the envelopes with the codes, and we irradiated the littermates on the same day and gave them half of the marrow from their respective donor each and decided not to give them post-graft immune suppression and see what happened. The 6 mismatched littermates all succumbed to GvHD or graft failure in the first two weeks. The matched ones all lived for at least 30 days before dying from acute, subacute, or chronic GvHD. From this we learned two things: one was that serologic matching was of the utmost importance but that was not enough, one had to develop technologies to prevent GvHD directed at minor histoincompatibility antigens. That experiment was the very first demonstration of an in vitro assay predicting outcomes of a transplant. Recruiting another technician, we set up an HLA (human leucocyte antigen) typing assay system including a mixed leukocyte culture technique, and later we recruited John Hansen to run the lab. Effie Petersdorf is continuing these typing studies as you know, and typing is now almost perfect with the development of molecular assays rather than serological and mixed leucocyte matching. That has been a very important influential part of our work.

MM: I guess there were no CROs who came and monitored you and asked if the dogs consented to being transplanted!

RS: No.

MM: You were not mandated to declare adverse events?

RS: We did, from the very outset. Our patients were clearly informed.

MM: I was talking about the dogs!

RS: Oh, the dogs! You mean the pet dog owners? We told them that there was a high risk but if it succeeded, they would get them back and all they needed to do was let us know what happened to the animals so that worked out really well.

MM: So, Rainer, you have trained hundreds of transplanters across the globe. Did you see yourself playing the role of influencing physicians in any way, moving towards this field of stem cell transplantation?

RS: Yes, I still remember most of the fellows and post-docs who came through the laboratory like the first European ones, Hans-Jochem Kolb from Munich and Éliane Gluckman, from Paris, and what I wanted to instill in these individuals was to perform experiments that were well thought out, that had clear and simple read-outs with no equivocation and to expect that 80% or more of these experiments would have a negative outcome. But then to not despair because when thinking about a negative result, you often come up with something very, very positive that leads you to the next experiment. I also taught them to be tenacious, not to be discouraged easily but to reflect on why things went the unexpected way. In biology, it is so complex, you are thinking out something in your mind, or, based on in vitro studies that something should work the same way in vivo, but often, as I said, it doesn’t come out that way. The main thing is to be honest with yourself, to accept the results for what they are and not to beautify them and to be self-critical. These are the things that I try to convey to the young colleagues. Frankly, to me, teaching these individuals to be good scientists is probably one of the more gratifying aspects of my work, apart from helping patients of course, which has been a very, very high priority.

MM: I can assure you that all of them are very, very grateful. So, we spoke about the initial research you did, the passion, and the famous department or sub-division of transportation. I am very interested in your relationship with the patient and how, by looking at particular diseases, like acute leukemia or aplastic anemia, has this influenced the sort of questions that you have chosen to ask in your lab and also in the clinical research at the bedside?

RS: As physician scientists here in Seattle, until 1998, we did 3 months as attending physicians in service and since then it has been 2 months and the third month was consultations, second opinions and so on. So, you are on the frontline for a good portion of the year and encountering patients with otherwise fatal diseases and you want to help them. From the very outset, we had a lot of patients with aplastic anemia which, in the pre-ATG era was an almost always immediately fatal disease, and so I spent a fair amount of time working on that problem. We developed in monkeys, the conditioning regimen that is still being used today, the 50 mg/kg/day × 4 days “cytoxan”, then later on, in dogs, we developed the addition of ATG. We were expecting rejections because of sensitization to minor histocompatibility antigens based on studies we published in the 1960s and 1970, and so we worked on figuring out how to overcome that. We eventually developed techniques to leucodeplete blood products and then found, serendipitously, that irradiating the blood product eliminated sensitization to minor antigens almost completely. We developed the ATG/cytoxan conditioning regimen that has now entered the mainstream. The next frontier was leukemia patients and of course GvHD prevention was a big item for all transplants but in leukemia, relapse was the big issue from the get-go. One of the problems was that early patients were all in full-blown refractory relapse and so the relapse incidence was extremely high. Transplanting patients in remission improved things but today relapse has remained the number one problem. We have a 5-year relapse rate of 35% and that almost always leads to death of the patient and so what drives me now is figuring out how to improve on this.

We cannot increase the intensity of the conditioning regimen, especially in older patients, so for the last 10 years our focus has been on using targeted radio-immunotherapy that has little general toxicity. We are fortunate to be able to work with our radiation physicists at the university here to develop an isotope called astatine-211, it has a short half-life, is very powerful and has a very short path length (~ 60 micron). Another colleague Scott Wilbur in the Radiation Oncology Department here, helped us with coupling it to any antibody using a boron cage and that’s now in a clinical trial with the hope of decreasing the relapse rate. It may not be enough and so we are currently working on a more innovative procedure which I can’t comment on yet because its just entering the pre-clinical experimentation stage. So, relapse is the big issue and it’s driven home to you when you transplant patients and then a year later, you hear that the patient has relapsed and that is almost always a death sentence and so I’m driven by the experiences I have with treating patients. It sends me back to the laboratory to try to figure out how to overcome this hurdle. I think that, given my age, it’s the last frontier that I want to still conquer.

MM: Maybe we will talk about this in a few years and celebrate the success of this innovation. Now we have heard several people mention your wife and your son. Can you say a few words about the contribution of your wife and the rest of your family during your career?

RS: My wife Beverly of course has had a tremendous influence on me. I met her in 1974, fortuitously, when she was a PhD student at the University of Pittsburgh in the Department of Genetics. A year later she got an NIH postdoctoral fellowship that enabled her to come to Seattle to work in the Division of Hematology. Beverley was always a very independent person and we always tried to keep our research separate from each other; we wouldn’t tell each other how to do experiments, although we did a few pieces of work together and have a number of joint publications. We co-mentored at least three postdoctoral fellows, one of whom was Maciej Zaucha who is now Head of Oncology at Gdansk University, another was Marco Mielcarek who is the Medical Director of the Adult Blood and Marrow Transplantation here at the Fred Hutch, and Professor at the University of Washington; the third was Paul Simmons who went to Australia. We support each other through the periods of time when things don’t work out in the laboratory, then we talk things through and give each other encouragement. I think the influence we exert on each other is more subtle and is not expressed in direct collaboration but thinking through failed experimentation and finding positive directions and helping each other that way.

My son is not in research, but we row together a lot, at least three or four times each week in the same boat, either the two of us in a double or with a couple of friends in a 4-person boat, so he and I are very close on that level. We do a lot of open-water races together, he is my favorite rowing buddy.

MM: So, he didn’t co-author any of the 1400 papers you have published so far! You have more than 100,000 citations and 152 have an *h*-index which is magic, I think, Rainer.

RS: I was unaware of that!

MM: Is there anything else that you would like to add at this time, especially something that you would have done differently in hindsight?

RS: No, not really, I consider myself really fortunate, firstly in having chosen my first postdoctoral fellowship in Paris, which apart from what I learned there in science, working with Jean Bernard and then Marcel Bessis and meeting Jean Dausset who worked in the same building and became a friend, I also experienced Paris in the early 60 s, which was a magical city for me coming from war-torn Germany. Then purely fortuitously, I made it to Seattle. I came for a period of three years and early on I had a lot of doubts on whether I would ever achieve anything with the work we were doing, to be quite frank, because we were so lonely, and experiments often didn’t work out. I can’t really overemphasize how lonely we felt, firstly, in this old hospital where we were the only researchers and secondly, in the whole world where there were only a handful of groups really interested in this topic of marrow transplantation. There were very few people to talk to, so the loneliness was amazing but then after three years I had a job offer in Germany with Ted Fliedner at Ulm University. I visited and by this time, we had made progress in the lab, and I could see a bit of light at the end of the tunnel and so I decided to decline the offer. Don said there was money for another year, was that okay? I said I’ll take my chances because I couldn’t apply for NIH research grants yet because I was still an alien. I only got my first NIH grant in 1971, on the same day that I got my green card. You couldn’t apply for NIH funding without the green card. I’m eternally grateful to the NIH for their continued support for that long time, regardless of the fact that NIH grants are hard to get and one is never certain whether the next competitive renewal application will get funded.

The other thing that happened was that Seattle began growing on me. I loved the environment, the water, mountains, I’m a climber, a skier and a rower, and I liked the people in the Pacific Northwest who are liberal and open-minded; I still like them! Seattle is one of my preferred places in the USA. So, I can’t really think of things I would have done differently. I happened to slide into these positions, haphazardly, although driven by this desire to work in a laboratory where I could do work that would be useful eventually to human patients, which was the main driver of staying here. I looked at a bunch of jobs in the USA, UCLA, Johns Hopkins, Universities of Pennsylvania, Michigan, Wisconsin, and many others, but I always came back to Don and said I’d looked at these jobs, there’s a lot of administrative stuff, university politics, could I stay here? He said sure, as long as I was willing to obtain research grant funding and didn’t mind it, I could stay forever, so, I think I did make the right decision. I think I was fortunate to have landed in this town with the help of this incredible Fulbright scholarship and the help of the NIH funding that has enabled me to work in science and medicine, and which will end in 2025 when I’m 90 years old! I will then stop working and that’s when my last program project grant will end.

MM: We heard a few minutes ago about hopefully, your successful future innovation to try to decrease the incidence of relapse after transplant. On a broader scale, I would say, what are your hopes today for the future of hematology?

RS: Well, there’s benign hematology, there’s malignant hematology and I think the latter is the more fascinating one with all these patients with leukemia, lymphoma, myeloma, myelodysplasia. The idea is that by trying to figure out what are the drivers of these malignancies maybe there is a way of switching them off. So far, unfortunately, that hasn’t panned out for any of the diseases. If we look at AML, we know that FLT3 is one of the drivers and we have FLT3 inhibitors and yet they don’t cure the disease. So, I’m hoping that we can make stem cell transplantation safer. We still haven’t entirely figured out how to prevent acute GvHD although we have got it down to a very low level of danger but we don’t know how to get rid of the chronic form so I hope that somehow, we can develop insight into how to turn that off. That’s what I’m banking on and perhaps somebody clever will figure that out. Once this has happened, I could imagine that transplantation would become a standard of care for a lot of these diseases. We have CAR T-cells, okay, but does this doesn’t always lead to a cure for all patients. Recently, the first CLL (chronic lymphocytic leukemia) patients were reported as living for 10 years without recurrence but those are still the exception rather than the rule. I’m hoping that by getting complete control of GvHD which has so far eluded us, this would enable standard of care stem cell transplants to be carried out in good non-university-based hospitals. The same is true for sickle-cell disease, we have gene therapy, but I was a little concerned about messing around with the hematopoietic system by introducing gene modifications, maybe CRISPR can do this, but I don’t actually know. I’m hoping that in the future, with better control of GvHD, we can let it exert the graft *versus* tumour effect and then turn it off. It will be somebody’s ingenious insight that will allow this and that’s what I’m hoping for.

MM: I have one last, but important question. What is your advice for the younger generation, if they should want to become a ‘giant’ like yourself?

RS: I guess you are talking about hematology/oncology fellows. I think the first thing is to pick a good mentor, somebody charismatic who you can connect with, discuss with, and also disagree with. Then, you have to be incredibly curious, curiosity is what drove me, and you must be tenacious, not easily be discouraged. You have to be able to take defeat and if you are an experimental worker, this comes almost on a daily basis and you must have the ability to think through why it failed, what when wrong, what can I learn from failure. Don’t be discouraged and don’t give up, take it on the chin and keep standing. Don’t shy away from getting your hands dirty. Those would be the important ingredients. Here in the USA, life is a little different to Europe where you have firm salaries, in the USA you have to be able to live with uncertainty. I wrote my very first grant in 1962, the NATO Science Fellowship application, to be able to work in Paris. Since December 1962 I have been working with the support of research grants and right now my salary is supported 50% by research grants, 25% by clinical income and 25% from the Center. You have to therefore have a boldness and think that you will be successful in getting grant funding and not having a guaranteed salary. Any young investigator must face these challenges in the USA, whereas in Europe things may be different with institutional or state set salaries.

MM: Well, I can’t add anything more except to say a big thank-you, unless you would like to add anything?

RS: No but I would like to thank you Mohamad for organising all of this. I really appreciate your friendship. I think you and I first met in Marseille if I’m not mistaken?

MM: Yes, almost 20 years ago.

RS: When I visited Didier Blaise and Christian Chabannon, and ever since then I have admired how you have handled yourself and have been able to occupy a very important position in French and European sciences, and so thank you for selecting me as your first candidate.

MM: I feel very humbled by your comments, and I did not select you it was a unanimous group decision so thank you again, it was a true privilege for me to moderate this first ‘standing on the shoulders of the giants’ and we are very grateful to the big giant, Dr Rainer Storb.

RS: Thank you.

